# Rosmarinosin A Inhibits Inflammatory Response in Lipopolysaccharide-Induced RAW 264.7 Macrophages via Suppressing NF-κB and MAPK Signaling Pathway

**DOI:** 10.3390/molecules30183752

**Published:** 2025-09-15

**Authors:** Hanui Lee, Gyeong Han Jeong, Seung Sik Lee, Kyung-Bon Lee, Sanghwa Park, Tae Hoon Kim, Hyoung-Woo Bai, Byung Yeoup Chung

**Affiliations:** 1 Advanced Radiation Technology Institute (ARTI), Korea Atomic Energy Research Institute (KAERI), Jeongeup 56212, Republic of Korea; hnlee11@kaeri.re.kr (H.L.); jkh4598@kaeri.re.kr (G.H.J.); sslee@kaeri.re.kr (S.S.L.); 2 Department of Radiation Science, University of Science and Technology (UST), Daejeon 34113, Republic of Korea; 3 Department of Biology Education, Chonnam National University, Gwangju 61186, Republic of Korea; kblee@jnu.ac.kr; 4 Bio-Resources Bank Division, Nakdonggang National Institute of Biological Resources (NNIBR), Sangju 37242, Republic of Korea; psh214@nnibr.re.kr; 5 Department of Food Science and Biotechnology, Daegu University, Gyeongsan 38453, Republic of Korea

**Keywords:** rosmarinosin A, anti-inflammatory, NF-κB, MAPK, dimeric phenylpropanoid

## Abstract

In the present study, we investigated the mechanisms underlying the anti-inflammatory effects of rosmarinosin A in (LPS)-stimulated RAW 264.7 macrophages. The cells were pretreated with various concentrations of rosmarinosin A, and then stimulated with LPS. Rosmarinosin A reduced the production of nitric oxide (NO) and prostaglandin E_2_ (PGE_2_), possibly through the modulation of inducible nitric oxide synthase (iNOS) and cyclooxygenase-2 (COX-2) expression, respectively. Additionally, it inhibited the production of pro-inflammatory cytokines, such as tumor necrosis factor α (TNF-α), interleukin (IL) 1β, and IL-6. The molecular mechanisms of rosmarinosin A involved the suppression of nuclear factor κB (NF-κB) p65 translocation into the nucleus. Furthermore, rosmarinosin A exhibited anti-inflammatory properties by suppressing the mitogen-activated protein kinase (MAPK) signaling pathway. These findings suggest that rosmarinosin A may exert its anti-inflammatory effects, at least in part, through the modulation of NF-κB and MAPK pathways in LPS-activated macrophages, offering the potential for therapeutic development.

## 1. Introduction

Macrophages are immune cells that are crucial for defending our bodies against infection and injury. However, when their activity goes unchecked, they can also fuel harmful inflammation, leading to various diseases [1]. Macrophages activated by various factors release pro-inflammatory cytokines such as tumor necrosis factor α (TNF-α), interleukin 1β (IL-1β), and IL-6. These cytokines, while helping fight infections, can also cause redness, fever, and tissue damage [2]. Two key enzymes, cyclooxygenase 2 (COX-2) and inducible nitric oxide synthase (iNOS), further amplify inflammation. COX-2 produces prostaglandin E_2_ (PGE_2_), which can suppress tumor cell death and promote angiogenesis, aiding tumor growth. iNOS generates large amounts of nitric oxide (NO), another inflammatory molecule [3]. Fortunately, several researchers are exploring ways to regulate these enzymes and curb excessive inflammation. Understanding the complex reactions of macrophages and their inflammatory mediators enables the development of new strategies to maintain health and combat chronic diseases [4].

Lipopolysaccharide (LPS), a component of bacterial cell walls, triggers a cascade of gene expression changes in immune cells, particularly monocytes and macrophages [5]. These changes, mediated by key signaling pathways like nuclear factor (NF) κB and mitogen-activated protein kinases (MAPKs), are crucial for mounting a proper immune response against infection [6]. However, excessive or dysregulated LPS-induced inflammation can lead to chronic inflammatory diseases such as arthritis and autoimmune disorders. NF-κB, a master regulator of inflammation, controls the expression of genes involved in immune response, such as adhesion molecules, chemokines, and MHC proteins [7]. LPS activates NF-κB through phosphorylation and subsequent degradation of IκB, resulting in the nuclear translocation of NF-κB. This, in turn, induces the transcription of pro-inflammatory mediators, including iNOS, COX-2, TNF-α, IL-1β, IL-6, and so on. MAPKs, representing another crucial signaling pathway, also contribute to LPS-induced inflammation [5]. They regulate the expression of COX-2 and iNOS, key enzymes involved in pro-inflammatory mediator production. Therefore, targeting both NF-κB and MAPKs holds immense potential for developing therapies to control excessive inflammation and its associated diseases.

Phenylpropanoids are a vast group of organic compounds found in plants. They are biosynthesized from the amino acids phenylalanine and tyrosine through the shikimic acid pathway [8]. These compounds play a crucial role in plant development and defense. They contribute to the structure of cell walls, protect plants from UV radiation or pathogens, and attract pollinators [8]. Additionally, phenylpropanoids possess a wide range of pharmacological activities, making them valuable for human beings [9]. Rosmarinic acid, a representative phenylpropanoid, is an ester of caffeic acid with dihydroxyphenyl-lactic acid and was first isolated and identified from rosemary (*Salvia rosmarinus*) in 1958 [10]. Furthermore, rosmarinic acid is one of the most abundant plant secondary metabolites found in food additives, herbal teas, and various medicinal plants. It has been extensively studied for its impressive range of biological activities, including antioxidant, antibacterial, and anti-allergenic properties, making it a promising candidate for various therapeutic applications [11,12]. Previously, we conducted research demonstrating that the radiolytic structural transformation of the phenylpropanoid rosmarinic acid led to the formation of the dimeric phenylpropanoid rosmarinosin A [13]. This compound was purified by chromatographic isolation. Rosmarinosin A is unique in that it contains a rare dihydrophenylpropanoid moiety, which was formed by the hydroxymethylation and cyclization of rosmarinic acid. This unique dihydrophenylpropanoid exhibited anti-adipogenic effects in 3T3-L1 preadipocytes [13]. However, research on the biological activity of rosmarinosin A is still very limited. In the present study, we investigated the anti-inflammatory activity of rosmarinosin A by focusing on the pro-inflammatory mediators in murine macrophages.

## 2. Results

### 2.1. Characterization of Rosmarinosin A

**Figure 1:**
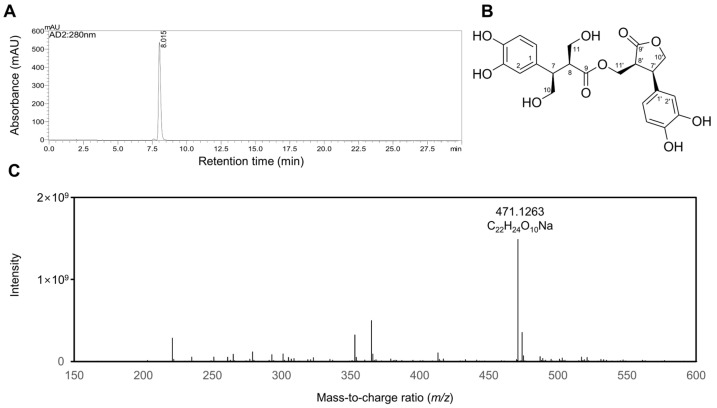
Characterization of rosmarinosin A. (**A**) HPLC chromatogram, (**B**) structure, and (**C**) HRESIMS spectrum of rosmarinosin A (RMA).

Pure rosmarinic acid dissolved in methanol was exposed to 50 kGy gamma irradiation. A high-performance liquid chromatography (HPLC) analysis of the irradiated solutions revealed a novel compound (See Supplementary Materials). The subsequent column chromatography of the irradiated mixture led to the successful isolation of the dimeric phenylpropanoid (Figure 1). The isolated compound was analyzed by HRESIMS, which showed a pseudomolecular ion peak at *m*/*z* 471.1263 [M + Na]^+^, consistent with the molecular formula C_22_H_24_O_10_. The ^1^H NMR spectrum (CD_3_OD) exhibited two ABX-type aromatic spin systems at *δ*_H_ 6.75 (1H, d, *J* = 1.8 Hz, H-2), 6.73 (1H, d, *J* = 7.8 Hz, H-5), 6.70 (1H, d, *J* = 7.8 Hz, H-5′), 6.67 (1H, d, *J* = 1.8 Hz, H-2′), 6.65 (1H, dd, *J* = 7.8, 1.8 Hz, H-6), and 6.54 (1H, dd, *J* = 7.8, 1.8 Hz, H-6′), indicating the presence of two 1,3,4-trisubstituted benzene rings. Signals for a dihydroxymethylated C_3_ moiety appeared at *δ*_H_ 4.50 (1H, t, *J* = 9.0 Hz, H-10), 4.12 (1H, t, *J* = 9.0 Hz, H-10), 3.93 (1H, dd, *J* = 11.4, 3.0 Hz, H-11), 3.66 (1H, td, *J* = 9.0, 3.0 Hz, H-7), 3.62 (1H, m, H-11), and 2.79 (1H, dt, *J* = 11.4, 3.0 Hz, H-8). Additional resonances attributable to a hydroxymethylated dihydrobenzofuran fragment were observed at *δ*_H_ 4.56 (1H, dd, *J* = 9.0, 6.6 Hz, H-10′), 4.47 (1H, dd, *J* = 9.0, 3.6 Hz, H-10′), 3.73 (1H, ddd, *J* = 8.4, 6.6, 3.6 Hz, H-7′), 3.58 (1H, dd, *J* = 10.2, 3.6 Hz, H-11′), 3.35 (1H, t, *J* = 10.2, H-11′), and 3.03 (1H, sept, *J* = 3.6 Hz, H-8′) (See Supplementary Materials).

The ^13^C NMR and HSQC spectra further supported this structural assignment, showing characteristic signals for a dihydroxymethylated propanoic acid unit at *δ*_C_ 179.6 (C-9), 73.9 (C-10), 58.7 (C-11), 51.1 (C-8), and 43.5 (C-7); a hydroxymethylated dihydrobenzofuran at *δ*_C_ 179.8 (C-9′), 74.6 (C-10′), 59.6 (C-11′), 48.5 (C-8′), and 44.7 (C-7′); and two trisubstituted aromatic systems at *δ*_C_ 146.9 (C-4), 146.8 (C-4′), 145.9 (C-3), 145.7 (C-3′), 131.1 (C-1), 130.6 (C-1′), 120.4 (C-6′), 119.9 (C-6), 116.8 (C-5′), 116.5 (C-2′), 115.9 (C-5), and 115.5 (C-2). Key HMBC correlations (H-11′ to C-9) established the linkage between the dihydroxymethylated C_6_–C_3_ fragment and the dihydrobenzofuran moiety. Furthermore, the small coupling constants (*J*_7,8_ = 3.0 Hz; *J*_7′,8′_ = 3.6 Hz), together with NOESY cross-peaks between H-7/H-8 and H-7′/H-8′, supported *erythro*-configurations at both stereogenic centers. Collectively, these spectroscopic results, in agreement with the literature data [13], identified the isolated compound as rosmarinosin A, a degradation product of rosmarinic acid.

### 2.2. Effects of Rosmarinosin A on LPS-Stimulated NO and PGE_2_ Production

**Figure 2:**
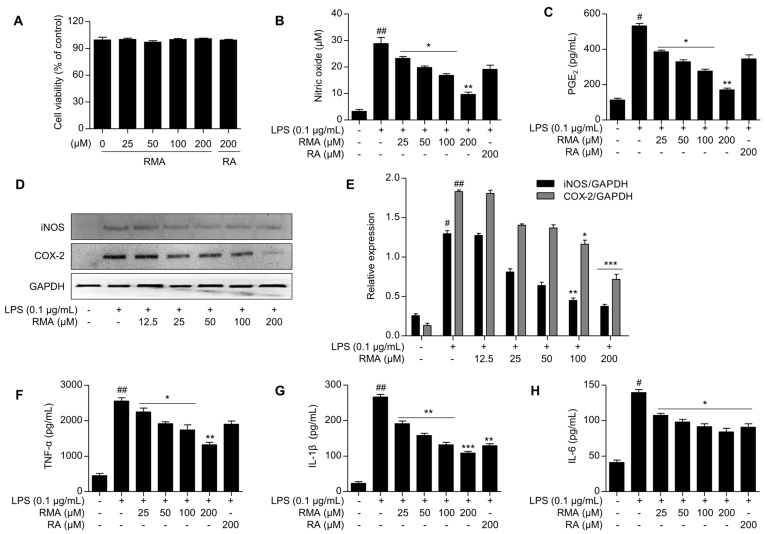
Effects of the rosmarinosin A on anti-inflammatory activities in LPS-stimulated RAW 264.7 cells. (**A**) RAW 264.7 macrophages were incubated with different RMA concentrations for 24 h. The cell viability was measured by the MTT assay. (**B**) The cells were pretreated with rosmarinosin A for 1 h and subsequently stimulated with 0.1 μg/mL for 24 h. The NO production was determined using the Griess reagent. (**C**) The PGE_2_ production was determined using ELISA. (**D**) The protein levels of iNOS and COX-2 in the cell lysates were analyzed by Western blotting. GAPDH was used as a loading control. The Western blotting data are shown as representative plots of three independent experiments. (**E**) The relative band intensity of each protein is expressed as a percentage. The (**F**) TNF-α, (**G**) IL-1β, and (**H**) IL-6 production was determined using ELISA. The results are expressed as the mean ± SD (*n* = 3). # *p* < 0.05 and ## *p* < 0.01 represent significant differences compared with the control group. * *p* < 0.05, ** *p* < 0.01, and *** *p* < 0.001 represent significant differences compared with only the LPS-induced group. RMA: rosmarinosin A, RA: rosmarinic acid.

First, to explore the anti-inflammatory effects of rosmarinosin A on LPS-stimulated RAW 264.7 macrophages, we investigated its potential effect on cell viability using the MTT assay. The results revealed that rosmarinosin A did not exhibit cytotoxicity up to a concentration of 200 μM (Figure 2A). This concentration was therefore chosen for the further investigation of rosmarinosin A’s anti-inflammatory properties.

To investigate the potential anti-inflammatory activity of rosmarinosin A, we employed RAW 264.7 macrophages, a well-established model, due to its ability to produce the inflammatory mediators NO and PGE_2_ upon LPS stimulation. Cells were pretreated with rosmarinosin A for 2 h before LPS (0.1 μg/mL) exposure for 24 h. The control groups received neither LPS nor rosmarinosin A. Subsequently, the NO production was quantified in the culture media using the Griess reagent. As expected, LPS stimulated a dramatic increase in NO generation from 3.2 ± 0.9 μM (control) to 28.8 ± 2.5 μM (Figure 2B). Rosmarinosin A induced a concentration-dependent reduction in NO production, indicating its anti-inflammatory potential. Furthermore, this effect was extended to PGE_2_ levels, with rosmarinosin A potently inhibiting LPS-stimulated production (Figure 2C). At the same concentration (200 μM), rosmarinosin A exhibited a stronger inhibitory effect on LPS-induced NO and PGE_2_ production compared to rosmarinic acid.

### 2.3. Effects of Rosmarinosin A on LPS-Stimulated Expression of iNOS and COX-2 Proteins

To further elucidate the mechanisms underlying the anti-inflammatory effects of rosmarinosin A, we examined the expression of the key inflammatory enzymes, iNOS and COX-2, using a Western blot analysis (Figure 2D). As expected, the LPS treatment significantly upregulated the protein levels of both iNOS and COX-2 in RAW 264.7 macrophages. However, pretreatment with rosmarinosin A reduced the expression of iNOS and modestly decreased the COX-2 levels in a concentration-dependent manner (Figure 2E).

### 2.4. Effects of Rosmarinosin A on LPS-Stimulated Pro-Inflammatory Cytokine Production

Figure 2F–H show the effects of rosmarinosin A on the production of key pro-inflammatory cytokines, TNF-α (Figure 2F), IL-1β (Figure 2G), and IL-6 (Figure 2H), in LPS-activated RAW 264.7 cells. The macrophages were treated with a range of rosmarinosin A concentrations (12.5 to 200 µM) and LPS (0.1 µg/mL) for 24 h. Rosmarinosin A reduced the production of pro-inflammatory cytokines, including TNF-α, IL-1β, and IL-6, in a concentration-dependent manner. Interestingly, while the TNF-α inhibition by rosmarinosin A was less pronounced, the suppression of IL-1β and IL-6 was comparable to that of the known anti-inflammatory compound rosmarinic acid.

### 2.5. Effects of Rosmarinosin A on LPS-Stimulated Expression of NF-κB and MAPK-Pathway-Related Proteins

**Figure 3:**
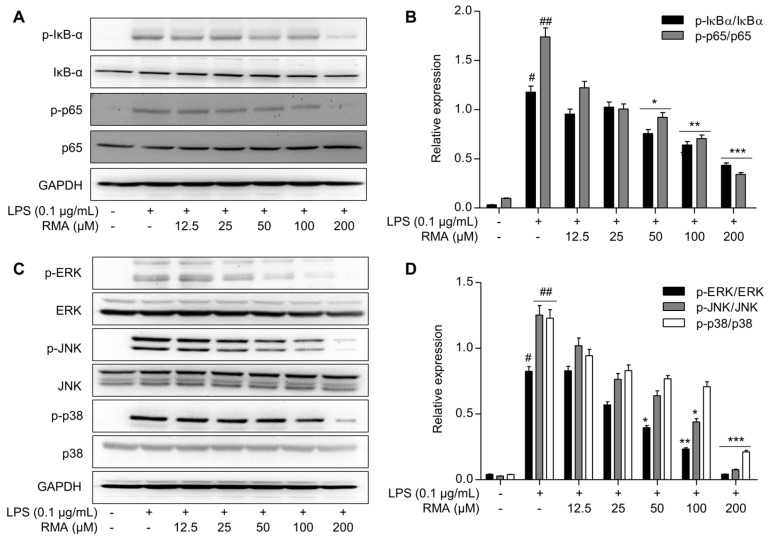
Effects of rosmarinosin A on the activation of NF-κB and the phosphorylation of MAPK in LPS-stimulated RAW 264.7 cells. The cells were pretreated with RMA for 1 h and subsequently stimulated with 0.1 μg/mL of LPS. (**A**) The protein levels of p-IκBα, IκBα, p-p65, and p65 in the cell lysate were analyzed using Western blotting. GAPDH was used as a loading control. The Western blotting data are shown as representative plots of three independent experiments. (**B**) The relative band intensity of each protein is expressed as a percentage. (**C**) The protein levels of p-ERK, ERK, p-JNK, JNK, p-p38, and p38 in the cell lysates were analyzed using Western blotting. GAPDH was used as a loading control. The Western blotting data are shown as representative plots of three independent experiments. (**D**) The relative band intensity of each protein is expressed as a percentage. The results are expressed as the mean ± SD (*n* = 3). # *p* < 0.05 and ## *p* < 0.01 represent significant differences compared with the control group. * *p* < 0.05, ** *p* < 0.01, and *** *p* < 0.001 represent significant differences compared to only the LPS-induced group. RMA: rosmarinosin A.

Further studies were conducted to determine the anti-inflammatory mechanisms of rosmarinosin A. As shown in Figure 3A,B, the LPS treatment markedly increased the levels of phosphorylated IκB-α and p65, indicating NF-κB activation (Figure 3A). Notably, treatment with rosmarinosin A (12.5 to 200 µM) significantly down-regulated the expression of both phosphorylated proteins in a dose-dependent manner (Figure 3B). These results indicate that rosmarinosin A may inhibit NF-κB activation by modulating IκB-α phosphorylation.

Next, we investigated the effects of rosmarinosin A on the MAPK signaling pathway to better understand the underlying mechanism. The Western blot analysis revealed that, while the total levels of the ERK, JNK, and p38 MAPKs did not significantly differ between the rosmarinosin A and LPS treatments compared with those of the controls, their phosphorylation levels (p-ERK, p-JNK, and p-p38) were significantly elevated in LPS-stimulated RAW 264.7 cells (Figure 3C). Importantly, the rosmarinosin A treatment led to a reduction in the phosphorylation levels of MAPK family members, suggesting a potential modulatory effect on this pathway. Specifically, the ratios of p-ERK/ERK, p-JNK/JNK, and p-p38/p38 were significantly decreased compared to those in LPS-treated cells (Figure 3D).

### 2.6. Effects of Rosmarinosin A on LPS-Stimulated NF-κB Transcriptional Activity via Nuclear Translocation of the p65 Subunit

**Figure 4:**
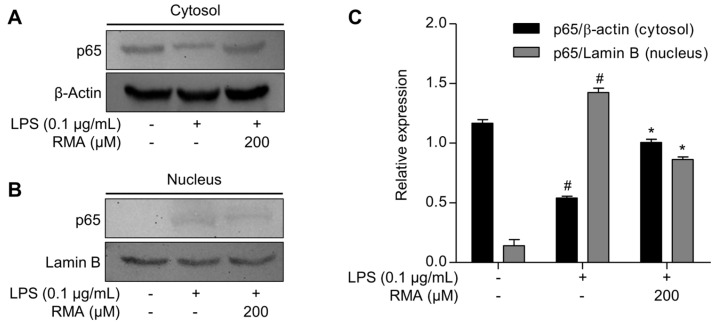
Effects of rosmarinosin A on the nuclear translocation of NF-κB p65 in LPS-stimulated RAW 264.7 cells. The cells were pretreated with RMA for 1 h and subsequently stimulated with 0.1 μg/mL of LPS. (**A**) The protein levels of cytoplasm p65 in the cell lysates were analyzed using Western blotting. β-Actin was used as a loading control. (**B**) The protein levels of nuclear p65 in the cell lysates were analyzed using Western blotting. β-Actin was used as a loading control. Lamin B was used as a loading control. The Western blotting data are shown as representative plots of three independent experiments. (**C**) The relative band intensity of each protein is expressed as a percentage. The results are expressed as the mean ± SD (*n* = 3). # *p* < 0.05 represents a significant difference compared with the control group. * *p* < 0.05 represents a significant difference compared to only the LPS-induced group. RMA: rosmarinosin A.

To further confirm the inhibitory effect of rosmarinosin A on NF-κB activation, we performed cytoplasmic and nuclear fractionation followed by a Western blot analysis to assess the intracellular localization of the total p65 protein. As shown in Figure 4A, the LPS treatment markedly decreased the p65 expression in the cytosolic fraction and increased it in the nuclear fraction, indicating nuclear translocation. However, pretreatment with rosmarinosin A (200 μM) significantly restored the cytosolic p65 levels and reduced its nuclear accumulation Figure 4B. The quantification of band intensities (Figure 4C) further confirmed that rosmarinosin A attenuates LPS-induced NF-κB nuclear translocation at this concentration, contributing to its anti-inflammatory mechanism.

## 3. Discussion

Natural products offer vast therapeutic potential; however, their limited availability often hinders their application. Rosmarinic acid is a representative dimeric phenylpropanoid constituent of many natural herbs, including rosemary (*S. rosmarinus* L.), perilla (*Perilla frutescens* L.), sage (*Salvia officinalis* L.), thyme (*Thymus vulgaris* L.), and peppermint (*Mentha* × *piperita* L.) [10]. Previously, it was reported that the rosmarinic acid content decreased when these herbs were irradiated with gamma rays at a dose of 10 kGy [14]. This study aimed to address this challenge by exploring new biologically active compounds derived from rosmarinic acid using gamma irradiation. Our previous study successfully identified four novel compounds from rosmarinic acid, including rosmarinosins A–C and (*S*)-oresbiusin A [13]. Although rosmarinosin A exhibits potent antiadipogenic activity, its broad potential remains unexplored. Therefore, this study investigated the anti-inflammatory activity of rosmarinosin A and potentially expanded its therapeutic applications. The discovery of these unexpected compounds highlights the potential of gamma irradiation in unlocking novel bioactive molecules from natural products, providing a valuable approach for drug discovery from natural sources.

Macrophages play a crucial role in immune defense, but the excessive production of pro-inflammatory mediators, such as NO and PGE_2_, can cause various inflammatory diseases. The regulation of NO and PGE_2_ production is a key strategy for the development of anti-inflammatory therapies [3]. In this study, we investigated the potential of rosmarinosin A, a novel compound derived from rosmarinic acid, to suppress the NO and PGE_2_ production in RAW 264.7 macrophages. Rosmarinosin A effectively suppressed NO and PGE_2_ production without exhibiting cytotoxic effects on the macrophages, suggesting its safety for potential therapeutic use. These findings suggest that rosmarinosin A is a promising candidate for further development as a novel anti-inflammatory agent.

The excessive production of NO and PGE_2_ by iNOS and COX-2 enzymes leads to various inflammatory diseases [3]. Chrysin and minaprine derivatives structurally modified by gamma irradiation effectively reduce iNOS and COX-2 expression [15,16]. In this study, we investigated the anti-inflammatory potential of rosmarinosin A, a novel dimeric phenylpropanoid derivative, by examining its effects on iNOS and COX-2 expression in LPS-stimulated RAW 264.7 macrophages. Our findings showed that rosmarinosin A reduced the NO and PGE_2_ production and attenuated the iNOS and COX-2 protein expression in a concentration-dependent manner, suggesting a potential modulatory effect on these inflammatory enzymes. These results suggest that rosmarinosin A exerts its anti-inflammatory effects by directly targeting the key biosynthetic enzymes responsible for NO and PGE_2_ production, a mechanism that could be further explored for potential pharmaceutical applications.

**Figure 5:**
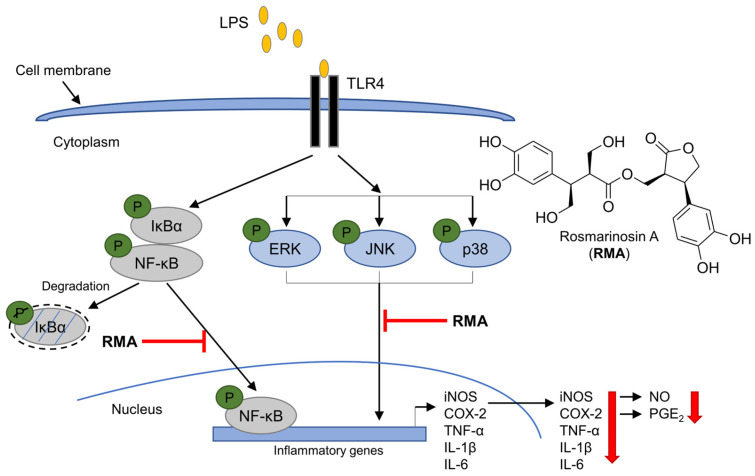
Schematic representation of the inhibition mechanisms by rosmarinosin A in LPS-induced inflammation. RMA: rosmarinosin A.

Abnormalities in cytokine production, such as TNF-α, IL-1β, and IL-6, are known to contribute to various inflammatory disorders [2]. TNF-α is a potent pro-inflammatory cytokine produced by immune cells, including monocytes and macrophages. It plays a key role in initiating and amplifying inflammation by stimulating the release of other inflammatory mediators, such as prostaglandins and chemokines. This can lead to tissue damage, pain, and swelling, which are hallmarks of many inflammatory diseases [17]. IL-1β is another major pro-inflammatory cytokine produced primarily by macrophages. It stimulates the production of other pro-inflammatory cytokines, increases the expression of adhesion molecules, and promotes the activation of immune cells. This cascade of events can contribute to tissue destruction and the development of autoimmune diseases, such as rheumatoid arthritis [18]. IL-6 is a complex cytokine with both pro- and anti-inflammatory properties. It is produced by various immune cells, including T cells, B cells, and macrophages. Its pro-inflammatory effects involve the stimulation of other pro-inflammatory cytokines and adhesion molecules [19]. Thus, the inhibition of cytokine production or function is a key mechanism in the control of inflammation. In the present study, we investigated the anti-inflammatory potential of rosmarinosin A by examining its effects on key cytokines in LPS-stimulated macrophages. We found that rosmarinosin A significantly suppressed the production of TNF-α, IL-1β, and IL-6 in a dose-dependent manner. These results demonstrate the potent anti-inflammatory activity of rosmarinosin A, further emphasizing its potential as a therapeutic agent for inflammatory conditions. Although the precise mechanism of action remains unclear, our findings suggest that rosmarinosin A may interfere with the pathways involved in cytokine production and signaling, possibly through upstream regulatory factors. Further studies are warranted to explore its efficacy in animal models of inflammation and elucidate the underlying mechanisms, including its potential interactions with other inflammatory mediators (Figure 5).

The NF-κB transcription factor family plays a critical role in inflammation. The inhibition of its activation has emerged as a promising therapeutic strategy to reduce severe inflammatory conditions, such as chronic inflammatory diseases and autoimmune disorders [6,7]. In addition to NF-κB, the MAPK pathways significantly contribute to the expression of genes involved in inflammation. When stimulated by bacterial components, such as LPS, through TLR4 receptors, proteins in these pathways are activated by phosphorylation and regulate specific transcription factors [20]. Therefore, both the NF-κB and MAPK signaling cascades are important targets for the development and production of effective anti-inflammatory substances. Our results revealed that rosmarinosin A partially inhibits the phosphorylation of MAPK proteins (ERK, JNK, and p38). In addition, it attenuated the phosphorylation of p65 and the degradation of IκBα, which are critical steps in NF-κB activation. We also confirmed that rosmarinosin A (200 μM) suppressed the nuclear translocation of NF-κB p65 in LPS-stimulated macrophages, as demonstrated by cytoplasmic and nuclear fractionation (Figure 4). These findings suggest that rosmarinosin A may influence both NF-κB and MAPK pathways, thereby contributing to its anti-inflammatory activity. However, further studies are required to clarify whether these pathways are modulated independently or through potential cross-talk.

## 4. Materials and Methods

### 4.1. Reagents and Antibodies

Anti-iNOS (#2977), anti-COX-2 (#4842), anti-ERK (#4695), anti-p-ERK (#4377), anti-p38 (#9212), anti-p-p38 (#9215), anti-JNK (#9252), anti-NF-κB (#8242), anti-p-NF-κB (#3033), anti-IκB (#4812), anti-p-IκB (#2859), anti-p-JNK (#9251), anti-GAPDH (#2118), anti-β-actin (#4967), and anti-lamin B1 (#15068) were purchased from Cell Signaling Technology, Inc. (Danvers, MA, USA). Dulbecco’s modified Eagle medium (DMEM), penicillin/streptomycin (P/S), and fetal bovine serum (FBS) were purchased from Lonza (Walkersville, MD, USA). The PGE_2_ and cytokine (TNF-α, IL-1β, and IL-6) kits were purchased from BD Biosciences (San Jose, CA, USA). The 3-(4,5-dimethylthiazol-2-yl)-2,5-diphenyltetrazolium bromide (MTT), Griess reagent, dimethyl sulfoxide (DMSO), and all other reagents were purchased from Sigma-Aldrich (St. Louis, MO, USA).

### 4.2. Preparation of Rosmarinosin A

Rosmarinic acid was obtained from Sigma-Aldrich (St. Louis, MO, USA). A 50 mg sample of rosmarinic acid dissolved in 50 mL of methanol was directly irradiated at 50 kGy in a Cobalt-60 irradiator (IIR-79, MDS Nordion International Co., Ltd., Ottawa, ON, Canada). High-performance liquid chromatography (HPLC) on a YMC-Pack ODS A-302 column (4.6 mm i.d. 150 mm, particle size 5 μm YMC Co., Kyoto, Japan) revealed the formation of rosmarinosin A alongside the starting material. The elution gradient started with 10% acetonitrile (MeCN) in 0.1% formic acid/water and increased to 100% MeCN over 30 min at 280 nm detection, a 1 mL/min flow rate, and a 40 °C oven temperature. The dried mixture (47 mg) was further purified by flash chromatography on an ODS AQ 120S-50 gel column (1.0 cm i.d. × 30 cm; particle size 50 μm; YMC Co.) using a 25% MeOH elution. Rosmarinosin A (5.5 mg, *t*_R_ 8.0 min) was isolated and the structure of rosmarinosin A was confirmed by a spectroscopic analysis (See Supplementary Materials).

Rosmarinosin A: Colorless oil, ^1^H NMR (CD_3_OD, 600 MHz): *δ* 6.75(1H, d, *J* = 1.8 Hz, H-2), 6.73 (1H, d, *J* = 7.8 Hz, H-5), 6.70 (1H, d, *J* = 7.8 Hz, H-5′), 6.67 (1H, d, *J* = 1.8 Hz, H-2′), 6.65 (1H, dd, *J* = 7.8, 1.8 Hz, H-6), 6.54 (1H, dd, *J* = 7.8, 1.8 Hz, H-6′), 4.56 (1H, dd, *J* = 9.0, 6.6 Hz, H-10′), 4.50 (1H, t, *J* = 9.0 Hz, H-10), 4.47 (1H, dd, *J* = 9.0, 3.6 Hz, H-10′), 4.12 (1H, t, *J* = 9.0 Hz, H-10), 3.93 (1H, dd, *J* = 11.4, 3.0 Hz, H-11), 3.73 (1H, ddd, *J* = 8.4, 6.6, 3.6 Hz, H-7′), 3.66 (1H, td, *J* = 9.0, 3.0 Hz, H-7), 3.62 (1H, m, H-11), 3.58 (1H, dd, *J* = 4.2, 10.2 Hz, H-11′a), 3.35 (1H, t, *J* = 10.2 Hz, H-11′), 3.02 (1H, sept, *J* = 3.6, H-8′), 2.79 (1H, dt, *J* = 11.4, 3.6 Hz, H-8); ^13^C NMR (CD_3_OD, 150 MHz): *δ* 179.8 (C-9′), 179.6 (C-9), 146.9 (C-4), 146.8 (C-4′), 145.9 (C-3), 145.7 (C-3′), 131.1 (C-1), 130.6 (C-1′), 120.4 (C-6′), 119.9 (C-6), 116.8 (C-5′), 116.5 (C-2′), 115.9 (C-5), 115.5 (C-2), 74.6 (C-10′), 73.9 (C-10), 59.6 (C-11′), 58.7 (C-11), 51.1 (C-8), 48.5 (C-8′), 44.7 (C-7′), 43.5 (C-7); HRESIMS *m*/*z* 471.1263 [M + Na]^+^.

### 4.3. Cell Culture

The RAW 264.7 mouse monocyte macrophage cell line (KCLB NO. 40071) was obtained from the Korean Cell Line Bank (Seoul, Korea). The cells were cultured at 37 °C in a humidified 5% CO_2_ atmosphere in Dulbecco’s modified Eagle medium (DMEM) supplemented with 10% fetal bovine serum (FBS) and 1% penicillin/streptomycin.

### 4.4. Cell Viability Assay

The cell viability of RAW 264.7 cells was assessed using the MTT assay [21]. The cells were seeded at 5 × 10^4^ cells/well in 96-well plates and incubated for 24 h at 37 °C. They were then treated with rosmarinosin A (12.5 to 200 μM) for 24 h in a serum-free medium. Subsequently, a 0.5 mg/mL MTT solution in DMEM was added to each well, and the plates were incubated for 3 h at 37 °C. Formazan crystals were dissolved in DMSO, and the absorbance was measured at 570 nm. The cell viability was determined relative to the control group (set as 100%).

### 4.5. Nitric Oxide (NO) Assay

RAW 264.7 cells (5 × 10^4^/well) were plated in a 96-well plate and incubated for 24 h at 37 °C. They were then pre-treated with various concentrations of rosmarinosin A for 2 h before LPS (0.1 μg/mL) stimulation for 24 h at 37 °C. The nitric oxide (NO) production was measured using the Griess reaction (Griess reagent). The culture supernatant (100 μL) was mixed with the Griess reagent (100 μL) at room temperature for 20 min. The absorbance of the reaction mixture was measured at 548 nm to determine the NO levels [22].

### 4.6. Enzyme-Linked Immunosorbent Assay (ELISA)

The PGE_2_ and cytokine production were determined by a sandwich ELISA. RAW 264.7 cells (5 × 10^4^ cells/well) were cultured for 24 h, and then treated with LPS (0.1 μg/mL) with or without rosmarinosin A (12.5 to 200 μM) for another 24 h. The supernatants were collected and the PGE_2_, TNF-α, IL-1β, and IL-6 levels were measured using specific mouse ELISA kits by following the manufacturer’s instructions. The sandwich ELISA principle utilizes capture and detection antibodies to quantify the target molecules.

### 4.7. Preparation of Nuclear and Cytosolic Extraction

RAW 264.7 macrophages were plated in 60 mm culture dishes at a density of 6 × 10^5^ cells/dish and incubated for 24 h. The cells were then pretreated with rosmarinosin A at concentrations of 200 µM for 1 h, followed by exposure to LPS (1 μg/mL) for 15 min. Following the treatment, nuclear and cytoplasmic extracts were obtained using the NE-PERTM Nuclear and Cytoplasmic Extraction Kit, according to the manufacturer’s protocol.

### 4.8. Western Blot Analysis

For the Western blot analysis, RAW 264.7 cells were lysed in RIPA buffer (containing protease and phosphatase inhibitors) to extract the proteins. Equal amounts (30–50 µg) were separated by 10% SDS-PAGE and transferred to PVDF membranes. After blocking with 5% non-fat dry milk, the membranes were incubated overnight at 4 °C with primary antibodies against specific target proteins at a 1:1000 dilution. Following washes with TBST, the membranes were incubated with horseradish peroxidase-conjugated anti-rabbit IgG secondary antibodies (Cell Signaling Technology, #7074) for 2 h at room temperature. Protein bands were visualized using the ECL chemiluminescent reagent (Millipore Corp., Billerica, MA, USA) and exposure to X-ray film (See Supplementary Materials).

### 4.9. Statistical Analysis

All the data were evaluated by a one-way ANOVA followed by Duncan’s multiple range test using SPSS 17.0 (SPSS Inc., Chicago, IL, USA), and the results were considered statistically significant when the *p*-value was <0.05. The experiments were performed at least three times independently. All statistical analyses were performed using GraphPad Prism ver. 8 (San Diego, CA, USA).

## 5. Conclusions

This is the first report concerning the mechanism of the anti-inflammatory activities of rosmarinosin A, generated from rosmarininc acid and modified by gamma-irradiation. In summary, rosmarinosin A potently inhibited the LPS-induced production of pro-inflammatory mediators (NO and PGE_2_) and cytokines (TNF-α, IL-1β, and IL-6) in RAW 264.7 macrophages. Furthermore, rosmarinosin A reduced iNOS and COX-2 protein expression in a dose-dependent manner. These effects may be mediated, at least in part, by the ability of rosmarinosin A to modulate NF-κB activation and MAPK phosphorylation. These findings suggest the potential of rosmarinosin A in developing anti-inflammatory therapeutics for acute and chronic inflammatory diseases.

## Data Availability

The datasets used and/or analyzed in the current study are available from the corresponding author upon reasonable request.

## Supplementary Materials

The following supporting information can be downloaded at: https://www.mdpi.com/article/10.3390/molecules30183752/s1, Figure S1: HPLC chromatograms of isolated rosmarinosin A. Figure S2: 1H NMR spectrum of rosmarinosin A in CD3OD. Figure S3: Expanded 1H NMR spectrum of rosmarinosin A (2.7–3.4 ppm). Figure S4: Expanded 1H NMR spectrum of rosmarinosin A (3.6–4.7 ppm). Figure S5: Expanded 1H NMR spectrum of rosmarinosin A (6.4–7.0 ppm). Figure S6: 13C NMR spectrum of rosmarinosin A in CD3OD. Figure S7: HSQC spectrum of rosmarinosin A in CD3OD. Figure S8: HMBC spectrum of rosmarinosin A in CD3OD. Figure S9: 1H-1H COSY spectrum of rosmarinosin A in CD3OD. Figure S10: NOESY spectrum of rosmarinosin A in CD3OD. Figure S11: Western blotting data for iNOS, COX-2, and GAPDH in LPS-stimulated RAW264.7 cells. Figure S12: Western blotting data for NF-κB and GAPDH in LPS-stimulated RAW264.7 cells. Figure S13: Western blotting data for MAPK and GAPDH in LPS-stimulated RAW264.7 cells. Figure S14: Western blotting data for cytosol and nucleus p65, β-actin, and lamin B in LPS-stimulated RAW264.7 cells.
