# Supplementary material for: Rosmarinosin A Inhibits Inflammatory Response in Lipopolysaccharide-Induced RAW 264.7 Macrophages via Suppressing NF-κB and MAPK Signaling Pathway
Source: Molecules. 2025 Sep 15;30(18):3752. doi: 10.3390/molecules30183752 (PMC12472359; doi:10.3390/molecules30183752)
Supplement: Supplementary file 1 [file molecules-30-03752-s001.zip › molecules-3802955-supplementary.pdf]

Supplementary Material

Rosmarinosin A Inhibits Inflammatory Response in Lipopolysaccharide-Induced RAW 264.7 Macrophages via Suppressing NF-κB and MAPK Signaling Pathway

Hanui Lee [1,†], Gyeong Han Jeong [1,†], Seung Sik Lee [1,2], Kyung-Bon Lee [3], Sanghwa Park [4], Tae Hoon Kim [5,*], Hyoung-Woo Bai [1,2,*] and Byung Yeoup Chung [1,*]

1. Advanced Radiation Technology Institute (ARTI), Korea Atomic Energy Research Institute (KAERI), Jeongeup 56212, Republic of Korea; hnlee11@kaeri.re.kr (H.L.); jkh4598@kaeri.re.kr (G.H.J.); sslee@kaeri.re.kr (S.S.L.)
2. Department of Radiation Science, University of Science and Technology (UST), Daejeon 34113, Republic of Korea
3. Department of Biology Education, Chonnam National University, Gwangju 61186, Republic of Korea; kblee@jnu.ac.kr
4. Bio-resources Bank Division, Nakdonggang National Institute of Biological Resources (NNIBR), Sangju 37242, Republic of Korea; psh214@nnibr.re.kr
5. Department of Food Science and Biotechnology, Daegu University, Gyeongsan 38453, Republic of Korea

* Correspondence: skyey7@daege.ac.kr (T.H.K.); hbai@kaeri.re.kr (H.-W.B.); bychung@kaeri.re.kr (B.Y.C.)

† These authors contributed equally to this work.

Contents

Figure S1. HPLC chromatograms of isolated rosmarinosin A.

Figure S2. ^{1}H NMR spectrum of rosmarinosin A in CD_3OD.

Figure S3. Expanded ^{1}H NMR spectrum of rosmarinosin A (2.7-3.4 ppm).

Figure S4. Expanded ^{1}H NMR spectrum of rosmarinosin A (3.6-4.7 ppm).

Figure S5. Expanded ^{1}H NMR spectrum of rosmarinosin A (6.4-7.0 ppm).

Figure S6. ^{13}C NMR spectrum of rosmarinosin A in CD_3OD.

Figure S7. HSQC spectrum of rosmarinosin A in CD_3OD.

Figure S8. HMBC spectrum of rosmarinosin A in CD_3OD.

Figure S9. ^{1}H-^{1}H COSY spectrum of rosmarinosin A in CD_3OD.

Figure S10. NOESY spectrum of rosmarinosin A in CD_3OD.

Figure S11. Western blotting data for iNOS, COX-2, and GAPDH in LPS-stimulated RAW264.7 cells.

Figure S12. Western blotting data for NF-κB and GAPDH in LPS-stimulated RAW264.7 cells.

Figure S13. Western blotting data for MAPK and GAPDH in LPS-stimulated RAW264.7 cells.

Figure S14. Western blotting data for cytosol and nucleus p65, β-actin, lamin B in LPS-stimulated RAW264.7 cells.

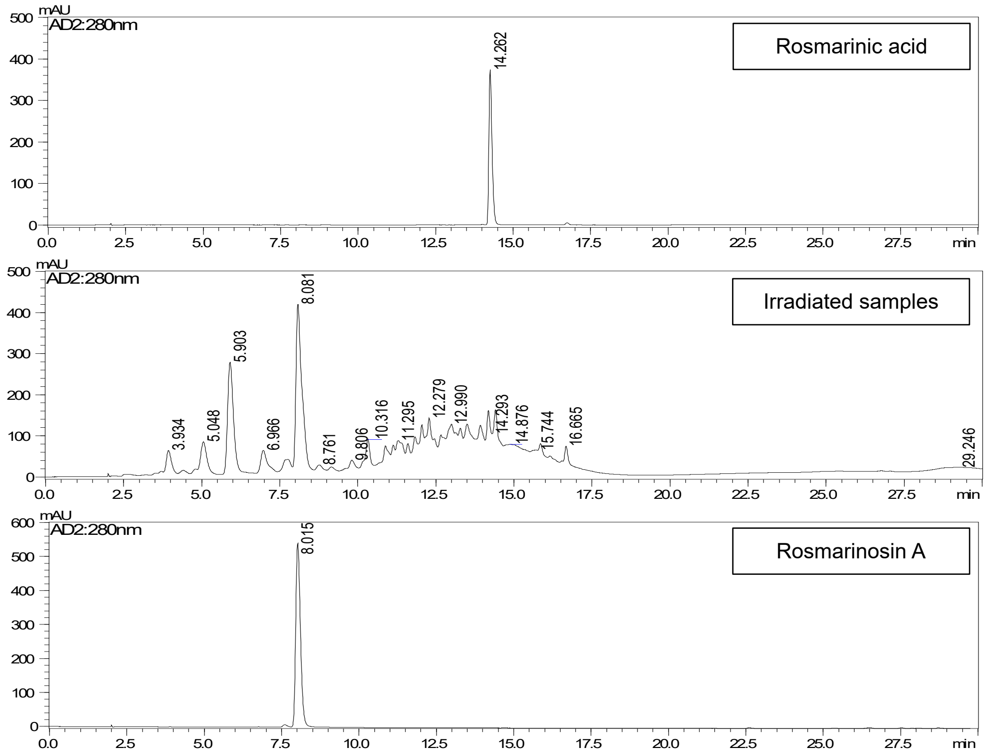

Figure S1. HPLC chromatograms of isolated rosmarinosin A.

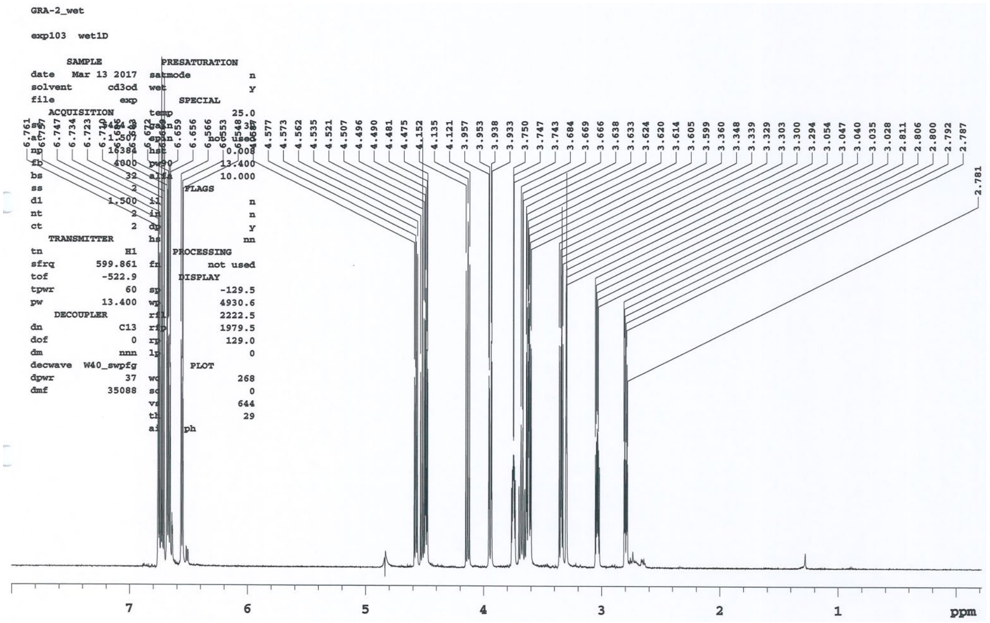

Figure S2. ^{1}H NMR spectrum of rosmarinosin A in CD_3OD.

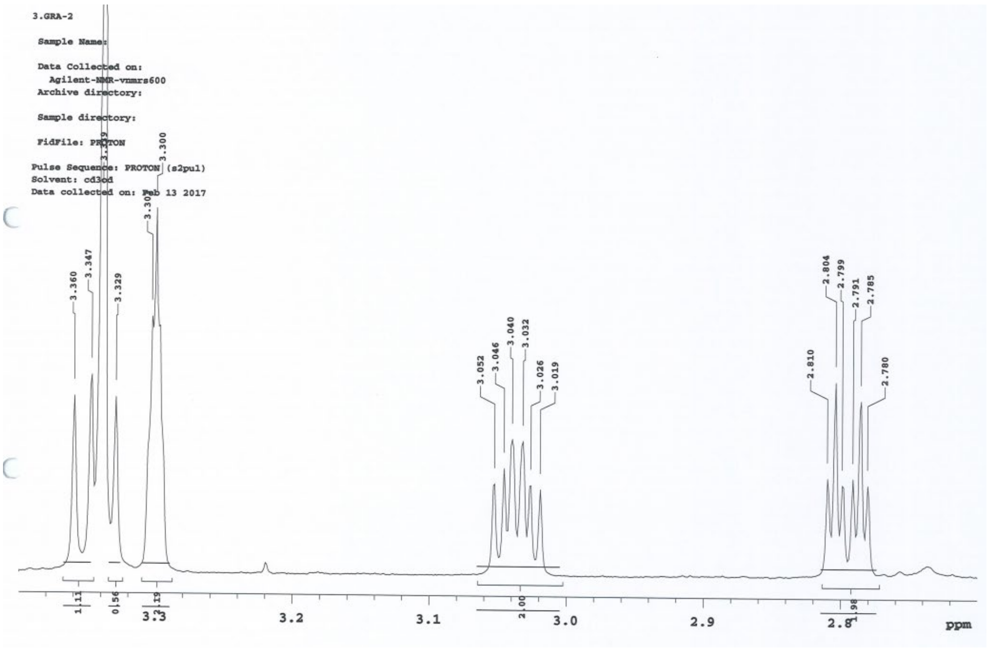

Figure S3. Expanded ^{1}H NMR spectrum of rosmarinosin A (2.7-3.4 ppm).

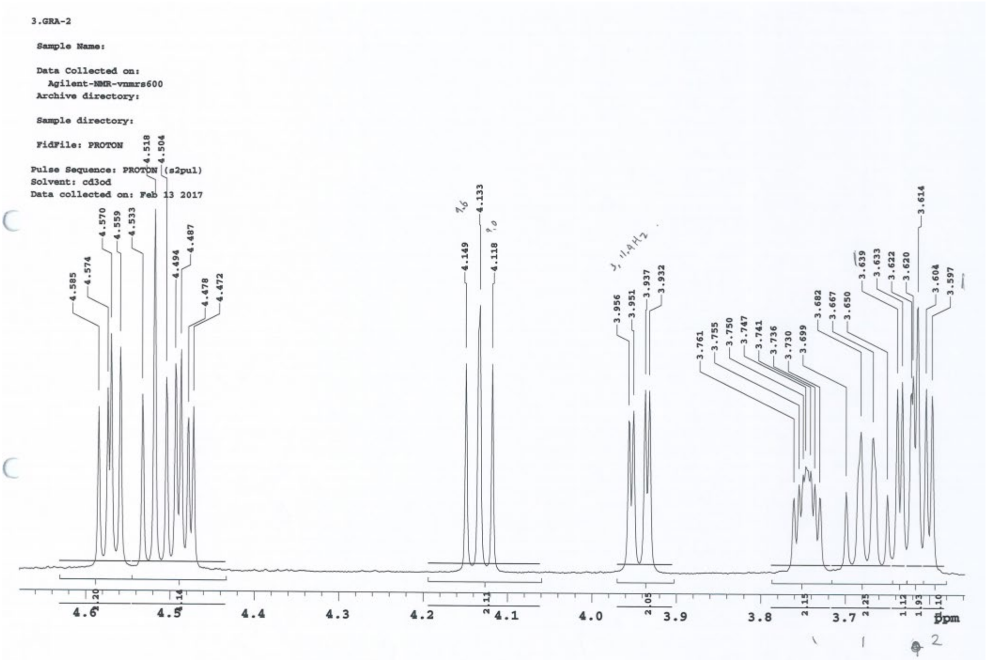

Figure S4. Expanded ^{1}H NMR spectrum of rosmarinosin A (3.6-4.7 ppm).

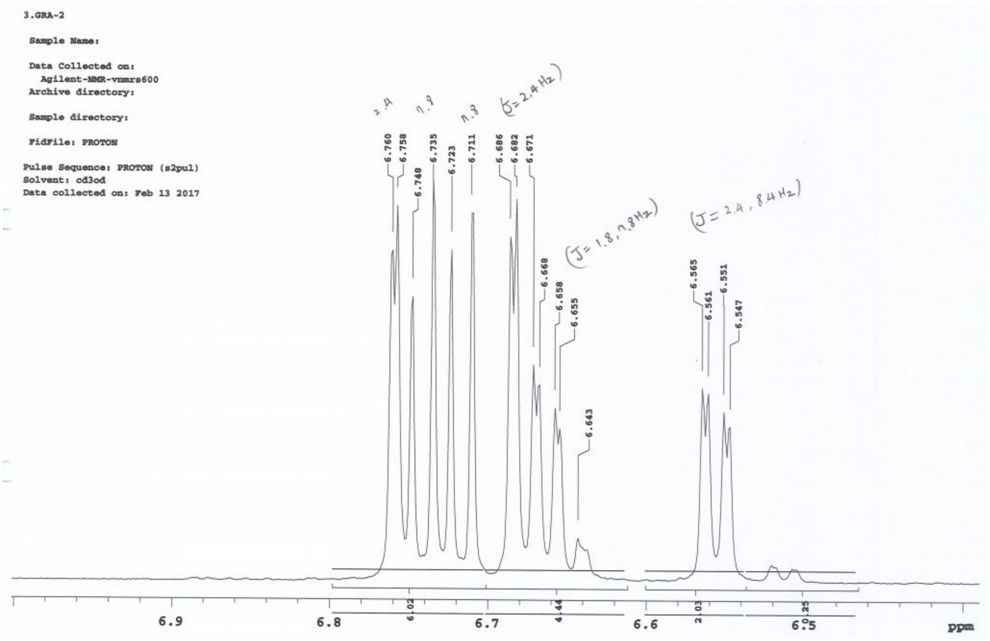

Figure S5. Expanded ^{1}H NMR spectrum of rosmarinosin A (6.4-7.0 ppm).

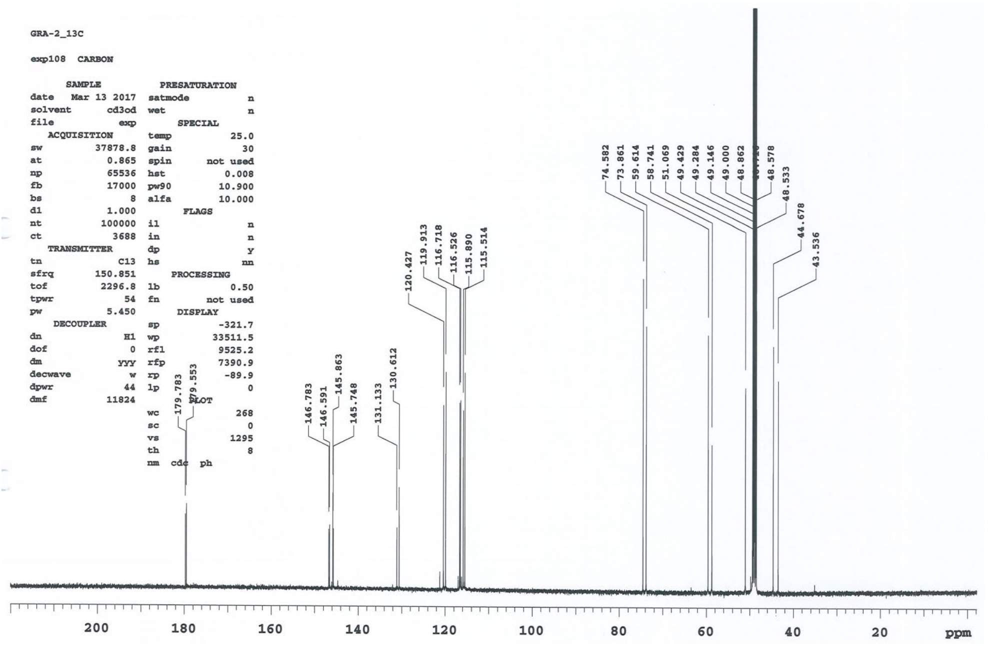

Figure S6. ^{13}C NMR spectrum of rosmarinosin A **2** in CD_3OD.

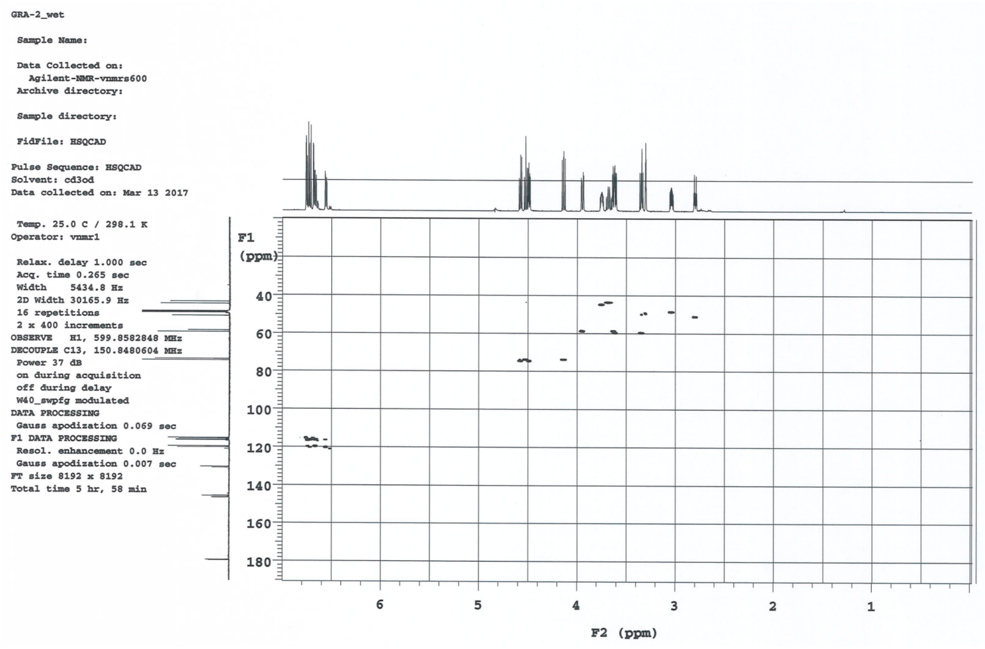

Figure S7. HSQC spectrum of rosmarinosin A in CD_3OD.

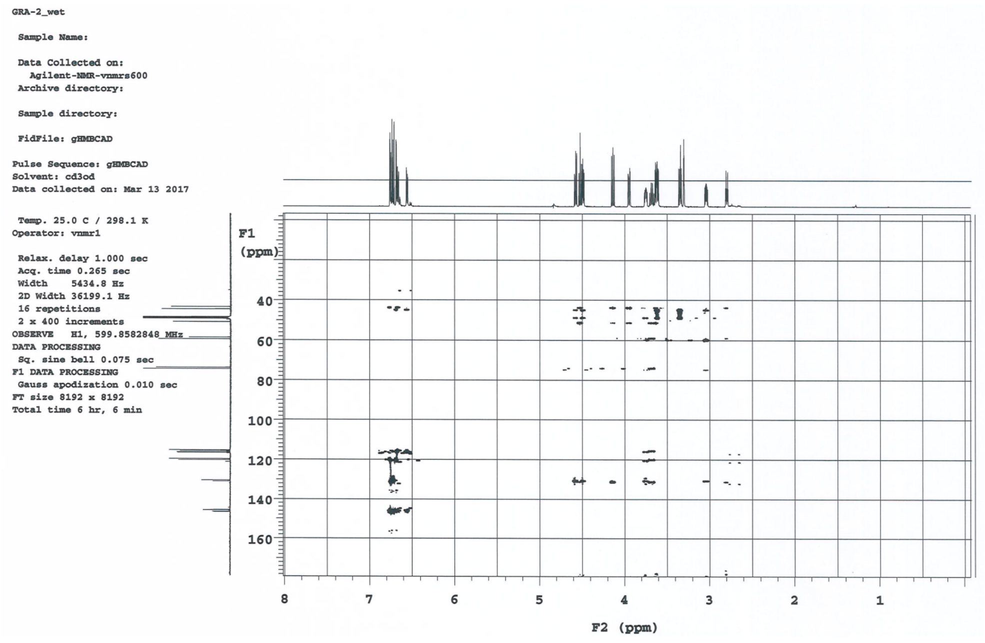

Figure S8. HMBC spectrum of rosmarinosin A in CD_3OD.

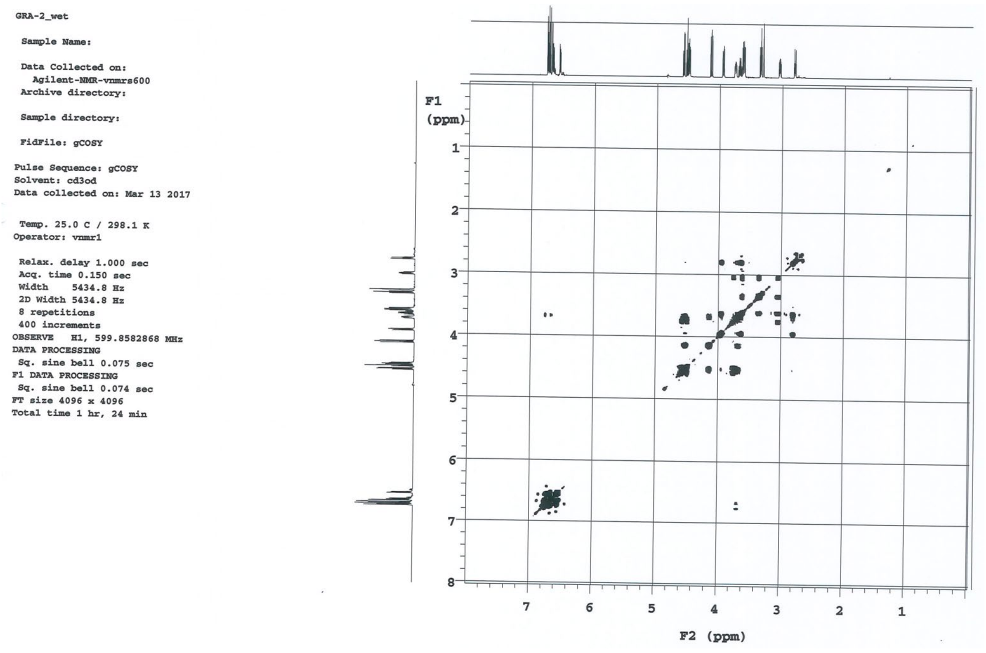

Figure S9. ^1H-^1H COSY spectrum of rosmarinosin A in CD_3OD.

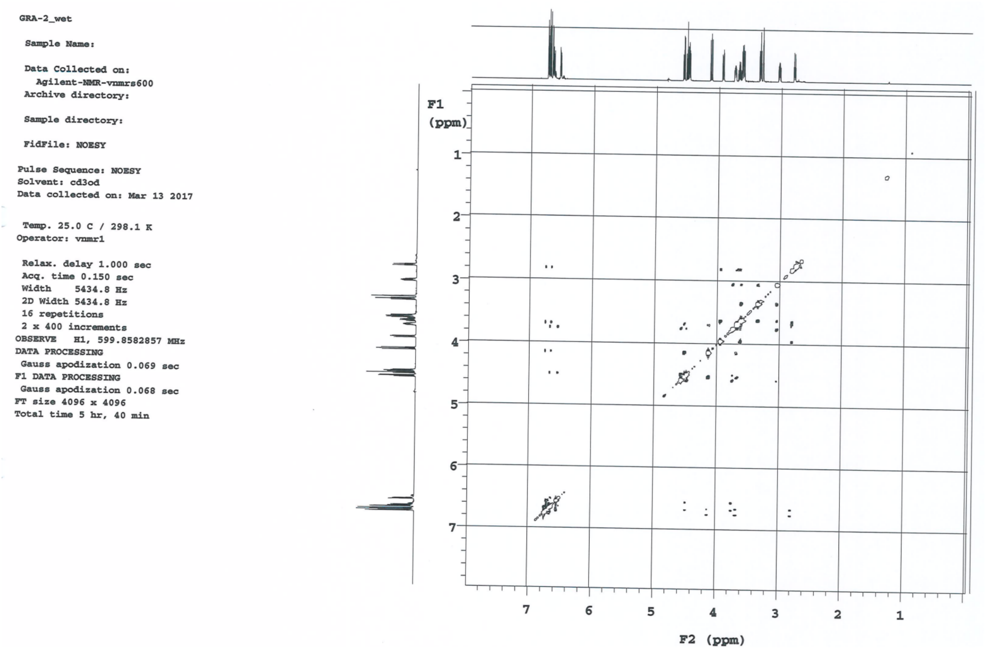

Figure S10. NOESY spectrum of rosmarinosin A in CD_3OD.

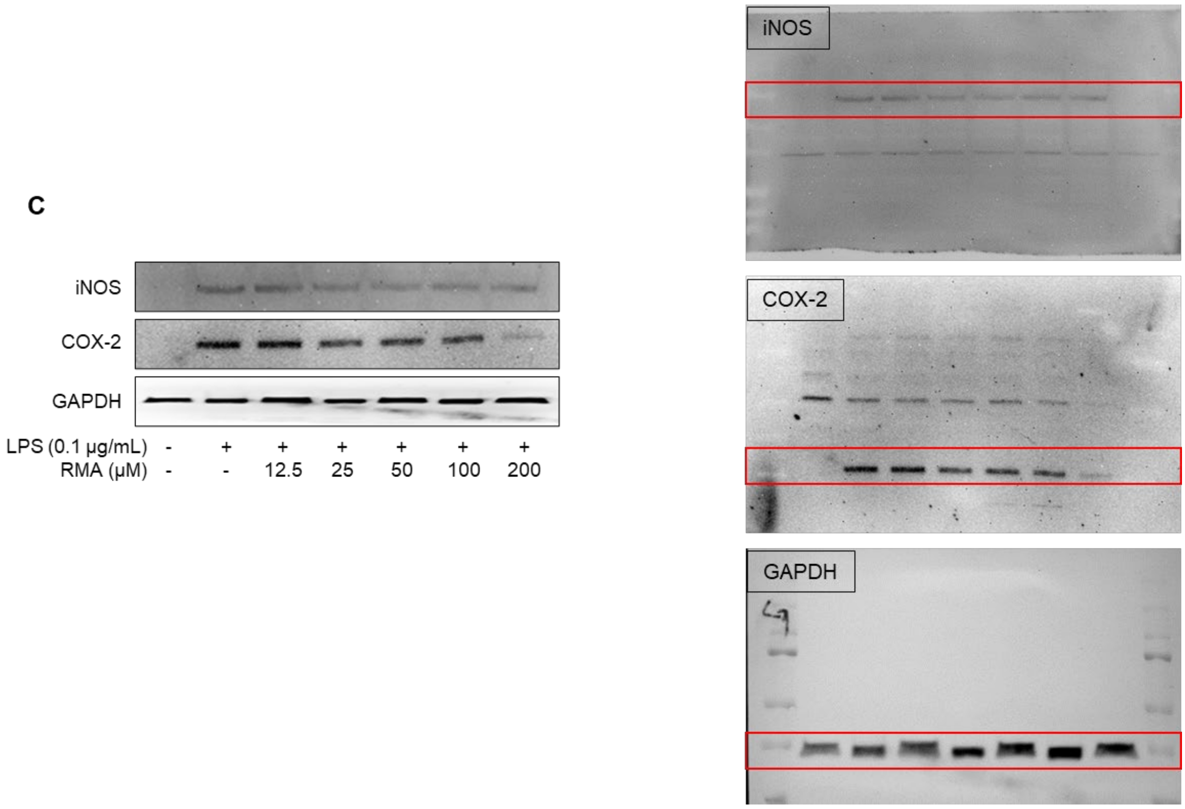

Figure S11. Western blotting data for iNOS, COX-2, and GAPDH in LPS-stimulated RAW264.7 cells.

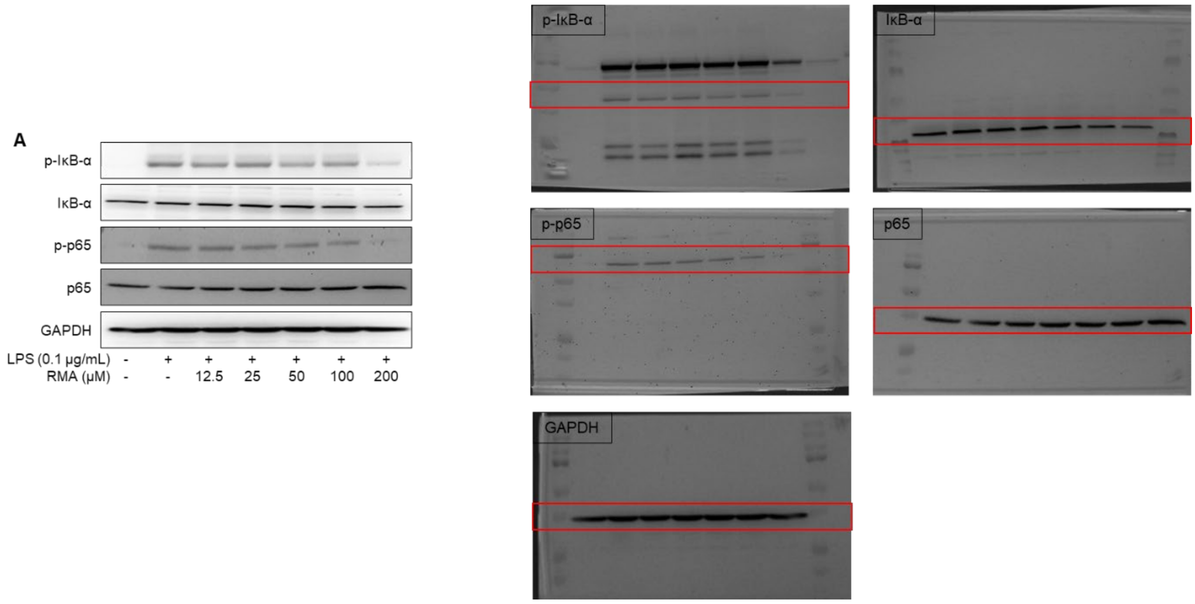

Figure S12. Western blotting data for NF-κB and GAPDH in LPS-stimulated RAW264.7 cells.

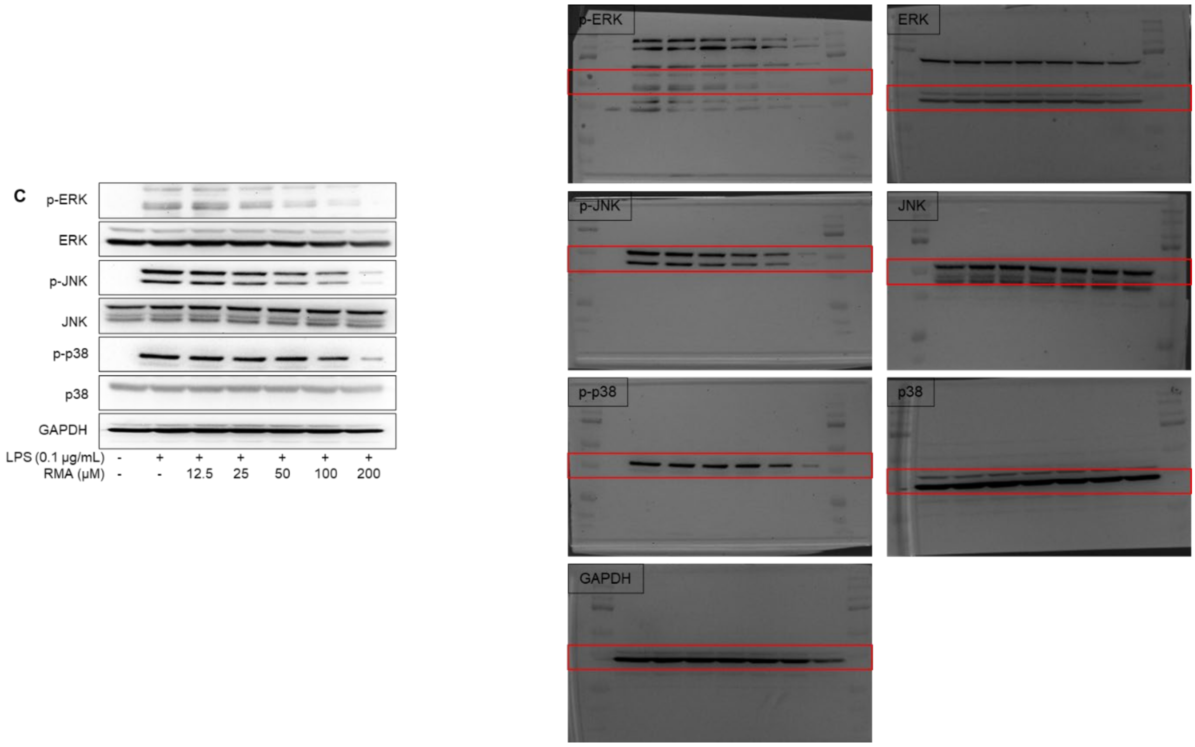

Figure S13. Western blotting data for MAPK and GAPDH in LPS-stimulated RAW264.7 cells.

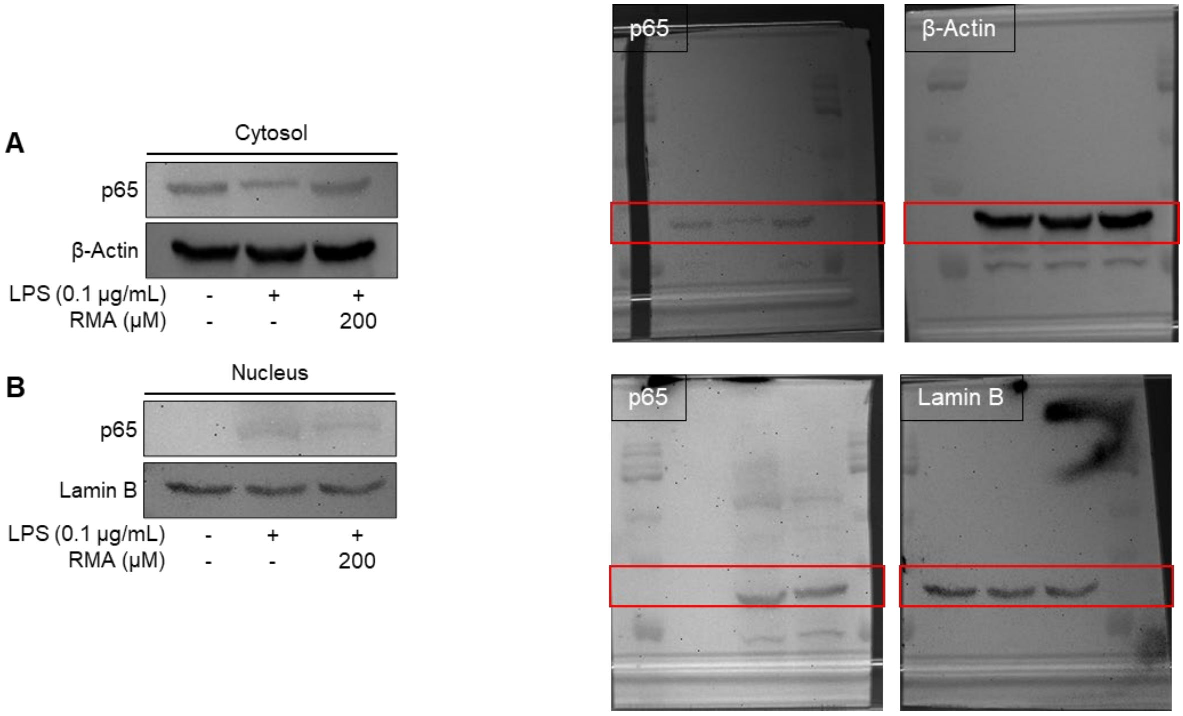

Figure S14. Western blotting data for cytosol and nucleus p65, β-actin, lamin B in LPS-stimulated RAW264.7 cells.
